# Biomechanical risk factors for ACL injury during a high-intensity exergame differ between the sexes based on exercise type

**DOI:** 10.1371/journal.pone.0324702

**Published:** 2025-05-21

**Authors:** Michelle C. Haas, Anna L. Martin-Niedecken, Larissa Wild, Leander Schneeberger, Eveline S. Graf

**Affiliations:** 1 School of Health Sciences, ZHAW Zurich University of Applied Sciences, Winterthur, Switzerland; 2 Department of Design, Zurich University of the Arts, Zurich, Switzerland; Erzurum Technical University: Erzurum Teknik Universitesi, TÜRKIYE

## Abstract

Incidence rates in anterior cruciate ligament injuries, which are linked to multiple factors, are higher in females than in males. Modifiable contributors to the difference in risk are biomechanical factors such as knee valgus and knee rotation or neuromuscular control. Despite considerable research efforts, re-rupture rates are still high necessitating the need for improved risk reduction and rehabilitation programs. Incorporating exergaming, physically active serious games including a dual-task, has high potential to address this need. However, the execution of dual-tasks leads to altered movement patterns. Consequently, a comprehensive understanding of these movement patterns and their sex-specific differences is essential to subsequently tailor the training approach. The aim of this study was to explore biomechanical differences between males and females when performing a high-intensive exergame. Using three-dimensional motion capture (Vicon) during a 25-min exergame (Sphery Racer, ExerCube), kinematics were measured in 18 healthy athletes (9 male, 9 female). Knee valgus, knee internal rotation, and hip flexion angles during 10–30° knee flexion, were compared between the sexes and in each of the nine different exercises. Touches, and punches showed significant sex differences for knee internal rotation angle (main effect of sex F(1,16) = 6.14, p = .025). Depending on the exercise and side, the difference in estimated means between males and females in touches and punches ranged from 4.6–7.8°, with females showing higher values. Therefore, females display distinct movement patterns linked to anterior cruciate ligament injury, indicating that these movements should be carefully integrated into routine training and late-stage post-injury rehabilitation.

## Introduction

Knee injuries are common among athletes in game sports and particularly in young athletes the rate of anterior cruciate ligament (ACL) ruptures is high [1,2]. Female athletes have an increased risk to sustain an ACL rupture compared to male athletes [2,3]. Multiple factors such as anatomy, hormones, movement patterns, or muscle activation patterns are commonly linked to a higher injury risk in females [4,5]. Although results of reviews support a multifactorial approach, only a subset of risk factors – such as neuromuscular or kinematic control – are modifiable and in need of more detailed investigation to reduce injury risk for females [6,7].

Biomechanical risk factors for ACL injury are derived from studying the non-contact injury mechanism. This mechanism involves a planted foot, a rotated tibia, an almost fully extended knee, and a valgus collapse of the knee when studied for deceleration or cutting and stopping activities [8–11]. Therefore, specific biomechanical risk factors have been identified and include an increased knee valgus (KV), increased knee internal rotation (KIR), decreased knee flexion, increased hip extension, and decreased ankle dorsiflexion [5,8,12–15]. Each of these factors contributes to the stress on the ACL which potentially could result in a rupture of the ligament. Moreover, it is likely that the injury mechanism is multiplanar necessitating the concurrent presence of biomechanical risk factors across various anatomical planes to induce an injury [13,14,16]. Understanding this multiplanar nature is crucial, as it highlights the complexity of ACL injuries and the need for comprehensive prevention strategies.

Decelerating while landing from a jump is a common injury situation [17,18], hence, biomechanical risk factors have often been investigated during landing tasks from a drop-jump [19,20]. Especially comparative analyses of landing mechanics in male and female athletes have been used to identify which biomechanical factors could be targeted in injury prevention programs for females. Compared to males, females land with increased peak KV and increased KIR, but smaller knee and hip flexion (HF) after both unilateral and bilateral jumping [19–23]. Current risk reduction exercises do not represent elements of the ACL injury mechanism well enough, especially multiplanar movements are lacking [24]. Hence, these factors should be targeted in injury prevention programs.

After ACL injury, the return to preinjury level of sports participation is only 63% in recreational athletes respectively 83% in elite athletes 13 months after injury, and fear of reinjury is high [25,26]. Additionally, rates of second ACL rupture are still high, evoking the need for improved rehabilitation programs, especially during late phases of rehabilitation [3,27,28]. In a narrative review, Buckthorpe (2019) suggests optimizations in the training of neuromuscular function, movement quality, sport-specific conditioning, and training load to improve late-stage rehabilitation [27]. Implementing these optimizations into rehabilitation requires more time in physiotherapy which could potentially strain the health system. However, incorporating modern technologies, such as virtual reality or serious games, has the potential to enhance current rehabilitation programs by incorporating multiplanar, explosive, sport-specific movements. Thus, allowing a high training load, biofeedback on performance, and motivational elements, which are all important aspects of rehabilitation [29]. Virtual reality rehabilitation or serious games are already used in motor rehabilitation of patients with acquired brain injury, Parkinson’s disease, multiple sclerosis, or cerebral palsy [30–32]. Exergames, defined as physically active serious games, require physical and cognitive effort and are increasingly used in rehabilitation for older adults, for example to reduce fall risk [33]. However, in sports rehabilitation, the use of exergaming is still limited [34], even though its increased physical fidelity, cognitive fidelity, and excitement (compared to traditional rehabilitation) has high potential [30].

Exergaming represents dual-task performance [35], hence, the cognitive resource theory can be applied. It states that only a limited amount of renewable cognitive resources is available necessitating a trade-off to manage these resources when performing a dual-task [36]. Therefore, either the motor or cognitive task is preserved while the other is limited. Results from previous studies investigating where cognitive resources are allocated to when a cognitive task is added to running in healthy participants, are inconclusive [37,38]. A study assessing dual-task effects in healthy participants while jumping concluded that the cognitive task impacted pre-landing biomechanics and preparatory neural mechanisms [39]. Therefore, it can be assumed that movement patterns of healthy participants are altered when exergaming. However, since previous dual-task performance studies used cognitive tasks such as the n-back test or word recall [37–39], and did not assess biomechanical risk factors for ACL injury, results cannot be directly transferred to motor behaviour while exergaming. Moreover, multiplanar movements during exergaming could represent the ACL injury mechanism better than existing exercises and could therefore fulfill the need for better risk reduction exercises [24].

Before applying exergames with patients, it should always be ensured that they meet the target group specific requirements (e.g., safety, specific movements, training load, etc.). Moreover, due to known differences between movement patterns in the lab or the field [40] and high ecological validity of the exergame, it can be expected that movement patterns will be altered when exergaming. However, if these alterations result in a higher load on the ACL is unclear. Given that squats and lunges do not result in high load on the ACL [12], they can be used as reference exercises with low risk for ACL injury. However, some of the exercises performed during exergaming the Sphery Racer in the ExerCube [41] – jumps, burpees, squats, lunges, touching or punching the wall – represent movements which are associated with ACL injury risk such as landing from a jump (jump and burpee) or cutting/side-stepping (touch and punch) [18]. Exercises are performed in quick succession to each other and the player has to move within the three-dimensional space of the exergame, hence, incorporating multiple planes. Moreover, given that exercises are performed oriented towards the front, right and left, thus coming from different sides into the next exercise, symmetry of the right and left leg cannot be assumed.

Since exergaming represents a dual-task and exercises may represent risky athletic motions for an ACL injury, biomechanical risk factors have to be investigated. It is known that males and females move differently during tasks related to ACL injury risk. Therefore, movement patterns of healthy males and females have to be compared in an exergaming setting to detect possible differences which could then be used to tailor training and rehabilitation with exergaming. Hence, the aim of this study was to explore differences in biomechanical risk factors for an ACL injury between males and females when performing a high-intensive exergame incorporating multiplanar movements. Based on current knowledge [19,20,42] we hypothesized that, compared to males, females show higher maximal KV (H1), higher maximal KIR angles (H2), and lower maximal HF angles (H3) during a high-intensive exergame training.

## Materials and methods

### Participants

Recruitment of a convenience sample took place between October 20^th^ 2021 and April 25^th^ 2022 via mail from the university campus and via flyer to regional sports clubs. To the best of the authors’ knowledge no prior research has investigated biomechanical movement patterns in a comparable setting. Consequently, a sample size calculation was not feasible and this study was conducted in an exploratory manner. Prior to enrolment in the study, participants were screened based on the following preselected inclusion criteria. Participants had to be between the age of 18 and 40 years, participate in a sports club (soccer, handball, volleyball, basketball, floorball, ice hockey, tennis, badminton, squash, or alpine skiing) at least three hours per week, and understand verbal and written instructions in German. Moreover, participants were excluded if they had a prior injury to the ACL, a prior knee surgery, acute or chronic musculoskeletal, neurological, or cardiopulmonary disease, severe pain during exercise execution, or were pregnant.

### Ethical considerations

This research was approved by the responsible medical Ethics Committee (Req-2021-01700) and complied with the tenets of the Declaration of Helsinki. Prior to data collection, written informed consent was provided by all participants. Participants had the right to withdraw from the study at any timepoint during the measurement session without stating a reason. The right to data privacy was ensured through two-factor authorization of trained study personnel when accessing data. Moreover, data was collected in an encoded form.

### ExerCube training

The ExerCube (Sphery AG, Au, Switzerland) is an exergaming device providing the Sphery Racer, an adaptive and functional high-intensity interval training experience [43]. While exercising, the player is surrounded by three walls to which the game is projected to such that the player feels physically immersed into the gaming environment. To interact with the system, the player wears a tracker (HTC Vive Tracker 2.0, HTC corporation, Taoyuan, Taiwan) on each wrist and ankle, which is used to track the player’s body position at any timepoint during the game. Nine different exercises (burpee, jump, squat, tripples, high-touch, mid-touch, low-touch, punch, lunge) are included and linked to specific ingame actions (e.g., overcoming targets on a sci-fi under water racing track) in the Sphery Racer. All participants played the Sphery Racer, whose exercises can be separated into side-specific exercises and neutral exercises [41,43,44]. Side-specific exercises involved moving from the center to either the right or the left wall, touching the wall, and moving back to the center, while in neutral exercises participants stayed in the center of the ExerCube. The high-touch, mid-touch, low-touch, punch, and lunge were classified as side-specific while the burpee, jump, squat, and tripples were considered as neutral exercises. Touches involved sidestepping to either the right or left wall and had to be performed at the visually indicated height. The three heights were above shoulder height (high-touch), at shoulder height (mid-touch), or below shoulder height (low-touch). Punches involved side-steps to the wall and a punch into the wall with the contralateral hand, e.g., participants performed side-steps to the right wall and punched into the wall with the left hand, thereby rotating the torso. For the lunge, a step forward either with the right or the left leg and in the direction of the corresponding corner was considered as a correctly performed lunge. Participants had to perform a squat, jump, or burpee in the center of the ExerCube and the exercises were symmetrically between the left and right side. While squatting, hands had to touch the floor, during jumps and jumps of the burpee, hands had to be streteched into the air. Tripples could be described best as running on the spot with synchronous arm and leg movements. However, as there is no relationship between ACL injury risk factors and running on the spot, tripples were excluded for analysis. As it is assumed that healthy athletes perform similarly to both sides, neutral exercises and side-specific exercises performed to the right wall were considered for analysis. However, movement is not symmetric and the right and left leg have to be considered separately [45]. First, all nine exercises were demonstrated and explained to the participants. For familiarization with the system, a 3-minute warm-up in the ExerCube including a tutorial was completed. After a short break, participants completed a 25-minute training in the ExerCube. The order of exercises was the same for all participants. Due to the adaptive nature of the ExerCube (game runs faster when movements are performed correctly), not all participants performed the same number of exercises (mean 630, standard deviation 43). Detailed information on the amount of performed exercises for each participant is available in the S1 File.

### Data collection and analysis

**Fig 1:**
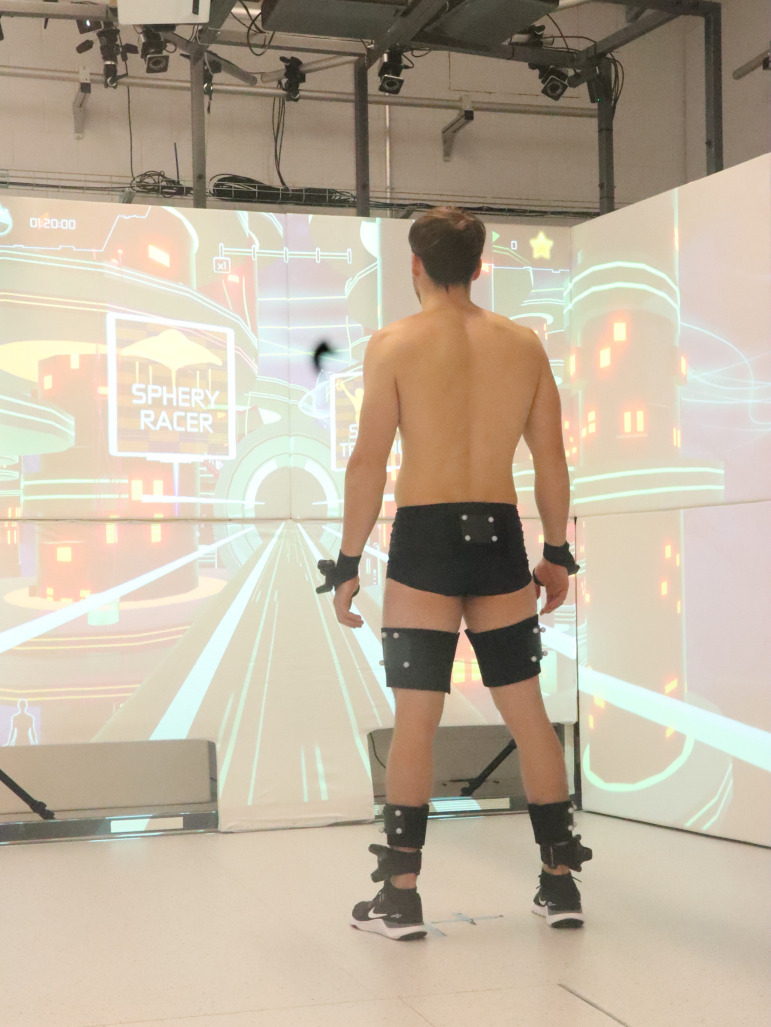
Measurement set-up.

The university’s movement laboratory was used for data collection. Marker clusters (B&L Engineering, Pinsco Inc, Santa Ana, United States) consisting of four markers on each cluster were attached to the lateral side of the thigh and shank on each leg and the posterior hip, similar to the cluster marker model of List et al. (2013) [46]. In total, 20 markers were attached. Measurement set up is displayed in Fig 1. A functional calibration task was used to locate the joint centres and joint angles of the knee and hip were calculated using a segmental approach [46]. Flexion, adduction, and internal rotation were assigned positive angular values. Data was collected using an infrared camera-based motion capture system operating at 240 Hz and including 11 cameras (Vicon, Oxford UK, Version 2.11). Under supervision of the investigator, every 60 seconds a new trial was automatically recorded and saved. Due to this approach, a few frames between trials could not be collected. Since the duration of the training was 25 minutes, 25 trials per participant were collected.

**Fig 2:**
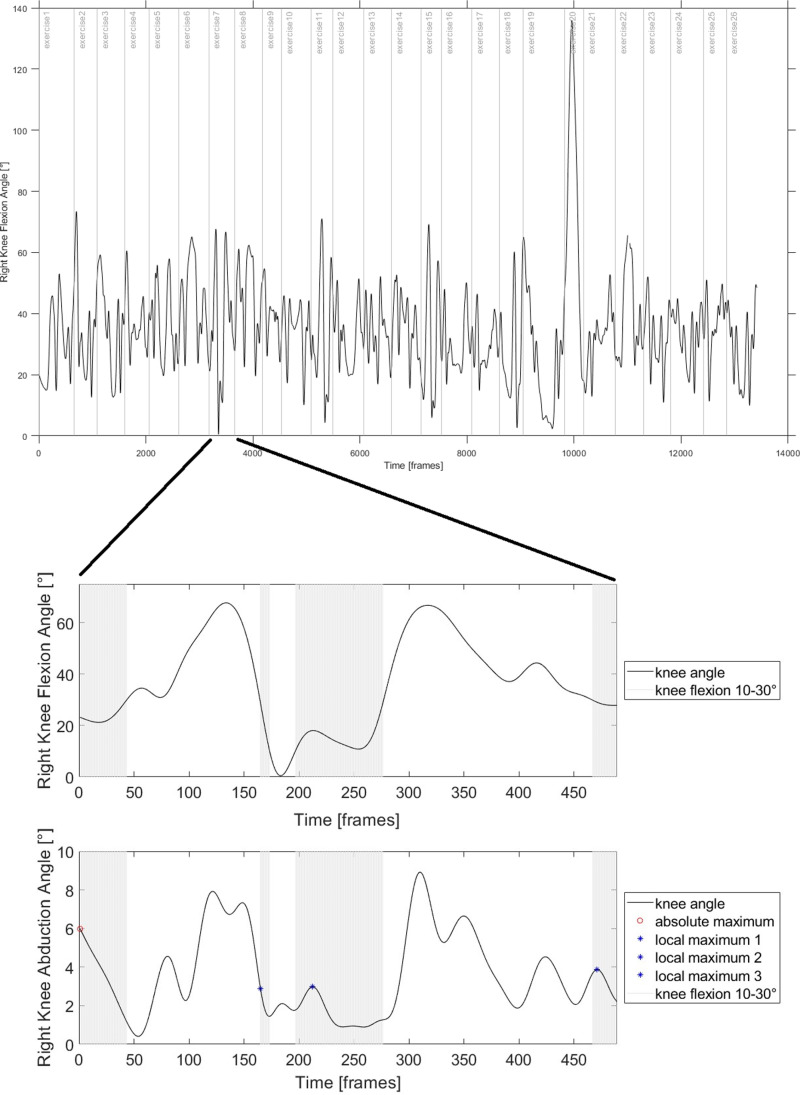
Illustration of the data processing and reduction procedure.

Post-processing of marker data was performed with MATLAB (MathWorks Inc. Natick, MA, USA, Version 2019a) applying a 4^th^ order Butterworth filter with cut off frequency of 7 Hz. The following stepwise approach, illustrated in Fig 2, was used to identify multiplanar biomechanically challenging movement patterns in each exercise. First, to have a single trial for each exercise, angle data in each 60-second-long trial was separated through video analysis at the most neutral position between exercises. The neutral position was defined as an upright trunk with both feet on the ground and straight legs. Side-specific exercises were labelled accordingly. When an exercise was not fully recorded (beginning or end of a 60-second-long trial) the exercise was excluded for analysis. Sliding of marker clusters resulted in exclusion of the corresponding leg (left leg, n = 1). Critical angles of the knee concerning ACL injury risk could occur at any point during the training, e.g., during an exercise or between two exercises, hence, data was neither cut nor normalized. For each exercise and body side, it was identified separately during which sections of the exercise the knee was between 10–30° knee flexion (= critical knee flexion angle, KF_crit_) which is related to high strain on the ACL [12]. Then, other biomechanical risk factors – maximal KV, maximal KIR, minimal HF – were extracted in all sections during which KF_crit_ occurred_._ For each exercise the maximal (KV, KIR) or minimal (HF) angle of all sections was used for further analysis. It is assumed that the strain on the ACL is highest when maximal KV, KIR, or minimal HF angles occur [47]. Outcomes (KV, KIR, HF) were analysed for both legs. For side-specific exercises, the right leg was labeled as ipsilateral and the left leg as contralateral.

### Statistical analysis

Participant characteristics between females and males were compared using Welch’s t-test. A linear mixed effects analysis of the relationship between sex (male or female), exercise, and side (right or left leg) was performed for data of each risk factor separately. This model allows to account for within-subject correlations due to the repeated measures design and allows for individual variability through random effects, hence, providing a flexible framework for an exploratory study. Side-specific exercises (high-touch, mid-touch, low-touch, lunge, punch) were differentiated from neutral exercises (burpee, squat, jump). Statistical analysis was performed using the packages lmerTest and lme4 in R Version 4.2.2 [48–50]. Fixed effects were sex, exercise, side, and their interaction. Random effects were intercepts for subjects. Using backwards optimization through likelihood ratio tests and the Akaike-Information-criterion, a final model was found for side-specific and neutral exercises. The aim of this procedure was to prevent overfitting of the data and ensuring optimal prediction for future data. Residual analysis through visual observation did not reveal any deviations from homoscedasticity or normality. P-values were obtained by restricted maximum likelihood tests and level of significance was set to *p* < .05. Likelihood ratio tests revealed that for side-specific exercises 2-way and 3-way interactions could not be removed, but in neutral exercises all interactions could be removed. The following model formula were used with $Y_{i}$ representing the outcome of interest, $\;\beta_{0}$ the intercept, $\beta_{k}$ the effect of the k-covariate, and $\varepsilon_{i}$ independent and normally distributed errors $\varepsilon_{i}\sim N(0,\;\sigma^{2})$.

Side-specific exercises:


$$Y_{ij}=\;\beta_{0}+\;\beta_{1}{side}_{j}*{exercise}_{j}*{sex}_{i}+\;\varepsilon_{i}$$

Neutral exercises:


$$Y_{ij}=\;\beta_{0}+\;\beta_{1}{side}_{j}+\;\beta_{2}{exercise}_{j}+\;\beta_{3}{sex}_{i}+\;\varepsilon_{i}$$

After analysis of the linear mixed model, marginal means were estimated and contrasts between the sexes were calculated using the emmeans package in R [51]. As no a priori sample size calculation was performed, results are of exploratory nature.

## Results

### Participant characteristics

Of the total 18 included participants, nine were male and nine were female. All participants were active members of a sports club in soccer (n = 7), floorball (n = 5), volleyball (n = 3), or handball (n = 3). Even though weight and height differed significantly between males and females, the body mass index and other characteristics did not (Table 1). The right leg was dominant in 16 participants (eight females) and the left leg in two participants (one female). One participant had prior experience with exergaming (one session in the Sphery Racer 1.5 years before the measurement session for this study).

**Table 1:** Characteristics of male and female participants, mean (SD).

Characteristic (unit)	Females (n = 9)	Males (n = 9)	Difference between sexes (Welch’s t-test)
Age (years)	24.9 (3.6)	25.6 (3.1)	T (16) = −0.42, *p* = 0.678, CI = (−4.01, 2.68)
Height (m)	1.7 (0.03)	1.8 (0.1)	T (12) = −5.79, *p* < .001, CI = (−0.17, −0.08)
Weight (kg)	63.2 (4.3)	74.9 (8.8)	T (12) = −3.56, *p* = .004, CI = (−18.79, −4.49)
BMI (kg * m^-2^)	23.2 (0.9)	23.6 (1.9)	T (12) = −0.63, *p* = 0.543, CI = −1.96, 1.09)
Sport-specific training (hours per week)	7.5 (3.4)	7.7 (3.7)	T (16) = −0.10, *p* = 0.921, CI = (−3.68, 3.35)
Number of exercises per ExerCube training	644.7 (50.9)	615.3 (30.6)	T (13) = 1.48, *p* = 0.162, CI = (−13.42, 72.10)

BMI = body mass index, SD = standard deviation, CI = confidence interval

### Side-specific exercises

In the side-specific exercises, the 3-way interaction (side, exercise, and sex) was significant in KV (F (4,7485) = 5.90, *p* < .001)) and KIR (F (4, 7485) = 15.26, *p* < .001)). 2-way interactions were significant in all investigated risk factors (interaction exercise and sex for KV *p* = 0.007, interaction side and sex for KIR *p* = .002, all other interactions with *p* < .001). A significant main effect of sex was found for side-specific exercises in KIR (F (1, 16) = 6.14, *p* = .025). However, the main effect of sex was neither significant in KV (F (1,16) = 1.36, *p* = .260) nor in HF (F (1,16) = 0.92, *p* = .352). In addition, main effects of exercise were found in KV (F (4, 7485) = 25.81, *p* < .001), KIR (F (4, 7485) = 92.60, *p* < .001), and HF (F (4, 7481) = 140.41, *p* < .001). Side showed significant main effects in KIR (F (1, 7486) = 19.13, *p* < .001) and HF (F (1, 7482) = 1589.94, *p* < .001), but not in KV. All differences between male and female athletes in exercises to the right wall are displayed in Table 2. There were no significant sex differences for HF in all exercises and sides. In total, 1541 high-touches, 1439 mid-touches, 1524 low-touches, 265 lunges, and 2748 punches were analysed. Descriptive results and the number of included exercises can be found in the S1 File.

**Table 2:** Differences between male and female athletes in knee valgus and knee internal rotation during side-specific exercises.

Exercise	Side	Knee valgus					Knee internal rotation				
		Estimated mean f [°] (95% CI)	Estimated mean m [°] (95% CI)	Difference (f-m) [°]	*p*	*d*	Estimated mean f [°] (95% CI)	Estimated mean m [°] (95% CI)	Difference (f-m) [°]	*p*	*d*
HT right	Ipsilateral	8.7 (7.3–10.1)	8.0 (6.6–9.4)	0.7	.482	.29	*13.7* *(10.9–16.4)*	*8.9* *(6.1–11.7)*	*4.8*	*.017*	*1.15*
	Contralateral	*9.5* *(8.1–11.0)*	*7.5* *(6.0–8.9)*	*2.0*	*.040*	*.84*	*13.6* *(10.8–6.4)*	*8.0* *(5.2–10.8)*	*5.6*	*.005*	*1.35*
MT right	Ipsilateral	8.9 (7.4–10.3)	7.7 (6.3–9.1)	1.2	.263	.46	*13.0* *(10.2–15.8)*	*7.2* *(4.4–10.0)*	*5.8*	*.004*	*1.39*
	Contralateral	*9.3* *(7.8–10.7)*	*7.2* *(5.8–8.6)*	*2.1*	*.042*	*.83*	*13.3* *(10.5–16.1)*	*7.9* *(5.1–10.7)*	*5.4*	*.008*	*1.29*
LT right	Ipsilateral	8.2 (6.7–9.6)	7.7 (6.2–9.1)	0.5	.635	.20	*10.9* *(8.1–13.6)*	*5.9* *(3.1–8.6)*	*5.0*	*.012*	*1.21*
	Contralateral	8.7 (7.3–10.1)	6.8 (5.4–8.2)	1.9	.059	.77	*11.9* *(9.1–14.6)*	*7.3* *(4.5–10.1)*	*4.6*	*.023*	*1.10*
PU right	Ipsilateral	9.0 (7.5–10.4)	7.9 (6.5–9.3)	1.1	.298	.43	*16.6* *(13.9–19.4)*	*8.8* *(6.1–11.6)*	*7.8*	*<.001*	*1.88*
	Contralateral	8.5 (7.1–9.9)	7.2 (5.8–8.6)	1.3	.207	.51	*12.8* *(10.0–15.5)*	*8.2* *(5.5–11.0)*	*4.6*	*.023*	*1.09*
LU right	Ipsilateral	6.9 (5.3–8.5)	6.3 (4.7–7.9)	0.6	.580	.27	9.2 (6.2–12.1)	5.8 (2.9–8.8)	3.4	.114	.81
	Contralateral	7.7 (6.1–9.3)	7.2 (5.7–8.8)	0.5	.643	.21	12.8 (9.8–15.7)	10.4 (7.4–13.4)	2.4	.272	.57

CI = confidence interval, f = females, m = males, HT = high touch, LT = low touch, MT = mid touch, PU = punch, LU = lunge, d = Cohen’s d effect size, *italics = p < .05*

### Neutral exercises

**Fig 3:**
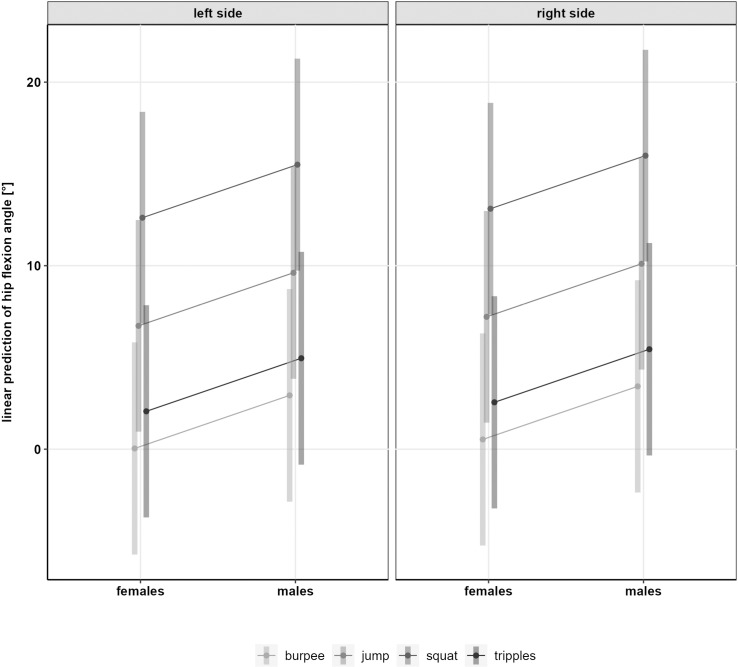
Linear prediction of the hip flexion angle in neutral exercises.

No significant differences were found between males and females in neutral exercises (KV: *p* = .982; KIR: *p* = .271; HF:*p* = .482). For KV (F(2, 35) = 62.70, *p* < .001), KIR (F(2,36) = 44.54, *p* < .001), and HF (F(3, 49) = 42.54, *p* < .001) an effect of exercise was found. In total, 2242 squats, 2850 jumps, and 487 burpees were used for analysis. In the S1 File descriptive results and the number of exercises per sex and side are provided. Estimated means for sex differences in KV and KIR are displayed in Table 3 whereas estimated means for non-significant sex differences in HF are displayed in Fig 3.

**Table 3:** Differences between male and female athletes in knee valgus and knee internal rotation during neutral exercises.

Exercise	Side	Knee valgus					Knee internal rotation				
		Estimated mean f [°] (95% CI)	Estimated mean m [°] (95% CI)	Difference (f-m) [°]	*p*	*d*	Estimated mean f [°] (95% CI)	Estimated mean m [°] (95% CI)	Difference (f-m) [°]	*p*	*d*
Burpee	Right	6.8 (5.3–8.3)	7.0 (5.5–8.6)	−0.2	.982	−0.12	10.8 (7.8–13.8)	8.6 (5.5–11.7)	2.2	.987	.63
	Left	6.8 (5.3–8.3)	7.0 (5.5–8.5)	−0.2	.982	−0.12	9.1 (6.1–12.1)	6.9 (3.9–9.9)	2.2	.987	.63
Jump	Right	5.8 (4.3–7.3)	6.0 (4.5–7.5)	−0.2	.982	−0.12	9.8 (6.8–12.8)	7.6 (4.6–10.7)	2.2	.987	.63
	Left	5.8 (4.3–7.3)	6.0 (4.5–7.5)	−0.2	.982	−0.12	8.1 (5.1–11.2)	6.0 (2.9–9.0)	2.1	.987	.63
Squat	Right	4.2 (2.7–5.7)	4.4 (2.9–6.0)	−0.2a	.982	−0.12	6.4 (3.4–9.4)	4.2 (1.1–7.2)	2.2	.987	.63
	Left	4.2 (2.7–5.7)	4.4 (2.9–5.9)	−0.2	.982	−0.12	4.7 (1.7–7.7)	2.5 (−0.5–5.5)	2.2	.987	.63

CI = conficence interval, f = females, m = males, HT = high touch, LT = low touch, MT = mid touch, PU = punch, LU = lunge, d = Cohen’s d effect size

## Discussion

The aim of this study was to explore differences between males and females in maximal KV, KIR, and HF within 10–30° of knee flexion during a high-intensive exergame. A significant main effect of sex was only found for KIR in side-specific exercises. Based on the statistics we cannot confirm H1 and H3 as there was no increased KV and decreased HF in females for all exercises. However, we can partially confirm H2 as side-specific exercises showed an increased KIR in female athletes. As no reference data using a similar approach is available and the study was of exploratory nature, discussion of the results is limited.

Contrasts between sexes for side-specific exercises revealed significant differences in the KV of the contralateral leg for the high-touch and mid-touch. Sex differences in KIR were found for the high-touch, mid-touch, low-touch, and punch in both legs. No significant sex differences were found in neutral exercises and HF. Significant differences of estimated marginal means of KIR between the sexes are bigger than 4.6°, with females showing significantly higher values. This could be explained by different movement strategies with females moving with more KIR, possibly in combination with internal rotation of the foot. We found smaller differences compared to existing literature investigating similar tasks like cutting, or sidestepping [19,52] and only in two exercises. This could be explained by the standardized task performance in existing literature, while in our study, task performance was not standardized thus allowing higher variability. Possibly, the dual-task during exergaming led to altered movement strategies in both sexes. High intra-subject movement variability within the 25-minutes of exergaming, either resulting from non-standardized task performance or performance of a dual-task, could mask bigger differences between the sexes [45]. Hip extension, which is associated with increased risk for ACL injury, occurred only in individual cases. To act as a protective factor for ACL injury, the hip has to be flexed by 30–40°, which is not the case in our results [12].

Due to interaction effects in side-specific exercises, the factors side and exercise impact differences in biomechanical risk factors for ACL injury risk between males and females. Thus, for each exercise and side, differences between males and females have to be considered separately. There are significant differences in both legs for touches and punches in KIR. Since only one participant of each group had a dominant left leg while in all the others the right leg was dominant, it is assumed that limb dominance did not play a role. In a healthy athletic population limb symmetry is above 94% during single-limb movements [53]. All touches (high, mid, and low) and punches performed to the right wall showed significantly higher angles in KIR, but not in KV, of females. However, confidence intervals for contrasts were high in all comparisons, possibly due to different movement strategies. A stable leg axis is important before the exercises can be incorporated into a rehabilitation-specific exergame. Our exploratory results suggest that especially in punches and touches the leg axis is not stable and should first be performed supervised and with high control outside of the exergame, avoiding the distraction of the exergame [54]. Once the leg axis is stable, touches and punches can be incorporated into training with the exergame. Then, especially for female patients, the focus during exergaming should be on the correct performance of touches and punches such that KV and KIR decreases.

Neutral exercises showed no differences between the sexes. In contrast, some existing literature shows that sex differences during the bilateral jump exist [55–57], while one other study also shows no sex differences [58]. Previous studies standardized the jump which was not the case in our study. Hence, greater within-subject variability could have affected statistical outcomes. Comparison of our results to literature investigating biomechanical risk factors at the knee during bilateral jumps, a 3.0° higher KV is found in males and a 4.6° lower KV is found in females in comparison to Hughes et al. (2008) [55]. However, compared to Swartz et al. (2005) the KV at initial contact of the jump is comparable (within a range of ± 2.2°) to our peak KV within KFcrit [58]. In general, our population showed a lower standard deviation indicating higher consistency than other studies. Even though both other studies investigated athletes trained in jumping and landing activities, differences could be a result of different training loads. For the KIR, no angles were reported in the aforementioned studies. For this reason, no reference data is available for KIR. Regarding HF, our sample showed no significant sex differences in HF as in Swartz et al. (2005) [58]. On a descriptive level, females had higher values at initial contact in Swartz et al. (2005), while our descriptive results show higher HF in males. Overall, HF was in a comparable range. Other studies investigating biomechanical risk factors for ACL injury during squats are available, but not comparable to our data since they investigated single leg squats and reported the peak range of motion which is much higher than KFcrit [59,60].

Discussion of the study results is limited because no reference data using a similar approach is available. Peak angles within KFcrit do not necessarily equal peak angles within the whole trial. In our view, KV, KIR, and HF during KFcrit better represent the multiplanar ACL injury mechanism than the peak angle during the whole exercise duration. Thus, with our results, we can add important insights to existing literature as well as to the future design of exergames for sports rehabilitation after ACL injury or its prevention.

Data collection in a real-world-like environment has advantages, such as better transfer to the practice, but also limitations. Limitations of this study were that neither exercises nor speed were standardized due to the real-world-like environment which could have resulted in different perceived intensities. Moreover, only a small sample size was included in this exploratory study. Participants performed a different number of exercises, but there was no significant difference in the number of exercises between the sexes. Given that the groups did not show any significant difference in key characteristics (age, BMI, hours of training per week), and all participants regularly engaged in game sports, it can be reasonably assumed that their athletic ability was comparable. Even though one participant had previous exposure to the exergame, we do not think that this impacted the movement pattern as exposure was only once and 1.5 years before the measurement session for this study. Due to the non-standardization of exercises, movement strategies could have differed between participants which led to high inter-subject variability of the data. The number of times an exercise was performed was not equal between exercises, for example burpees were performed rarely while jumps were performed frequently (S1 File), which limits the comparison between exercises. Though, the exercise sequence was fixed, and every participant had a similar number of each exercise. Another limitation of this study is that fatigue was not accounted for. Results might have been different if, for example, only the first and last minutes of the training duration would have been analyzed. The small sample size is a limitation and results might not be applicable to the general population. Even though marker clusters were secured with adhesive tape and markers did not move on the marker cluster, it is likely that there were small movements of the marker cluster on the skin, especially due to the highly dynamic nature of movements, which reduces accuracy. When cluster movement was detected, the data of the corresponding leg was excluded. Moreover, as the accuracy of determining the knee valgus and knee internal roation angle could have been limited, it is possible that the differences lay within the range of measurement error.

## Conclusion

In summary, sex differences in knee biomechanics during high-intensity exergaming appear to be most evident in knee rotation angles, and during specific exercises. Since females showed higher maximal knee angles than males, their individual movement strategies should be carefully observed before incorporating these exercises into high-intensity training to ensure movement strategies do not result in increased risk for knee injury.

## Supporting information

S1 File Descriptive results. (DOCX)
