# Supplementary material for: Biomechanical risk factors for ACL injury during a high-intensity exergame differ between the sexes based on exercise type
Source: PLoS One. 2025 May 21;20(5):e0324702. doi: 10.1371/journal.pone.0324702 (PMC12094794; doi:10.1371/journal.pone.0324702)
Supplement: S1 File — (DOCX) [file pone.0324702.s001.docx]

# Supplementary Material: Descriptive Results

Table 1: descriptive means, standard deviation, and number of observations of males and memales per side and neutral exercise

| Maximal knee valgus angle [°] | Females | | | | | | | Males | | | | | |
| --- | --- | --- | --- | --- | --- | --- | --- | --- | --- | --- | --- | --- | --- |
|  | Left | | | Right | | | | Left | | | Right | | |
| Exercise | Mean | SD | N | Mean | SD | N | Mean | | SD | N | Mean | SD | N |
| Squat | 4.51 | 3.2 | 572 | 3.91 | 3.73 | 594 | 4.04 | | 2.34 | 497 | 4.73 | 3.15 | 579 |
| Jump | 6.16 | 2.84 | 741 | 5.41 | 3.02 | 747 | 5.23 | | 2.20 | 628 | 6.46 | 2.96 | 734 |
| Burpee | 7.54 | 2.57 | 123 | 7.02 | 2.92 | 162 | 6.06 | | 1.93 | 97 | 6.64 | 2.79 | 105 |

*Note. SD = standard deviation, N = number of observations*

Table 2: descriptive means, standard deviation, and number of observations of males and females per side and side-specific exercise

| Maximal knee valgus angle [°] | Females | | | | | | Males | | | | | | |
| --- | --- | --- | --- | --- | --- | --- | --- | --- | --- | --- | --- | --- | --- |
|  | Ipsilateral | | | Kontralateral | | | Ipsilateral | | | Kontralateral | | | |
| Exercise | Mean | SD | N | Mean | SD | N | Mean | SD | N | Mean | SD | N |  |
| High-touch right | 8.8 | 3.3 | 411 | 9.6 | 3.7 | 414 | 8.0 | 3.2 | 379 | 7.2 | 2.6 | 337 |  |
| Mid-touch right | 8.9 | 3.2 | 384 | 9.3 | 3.7 | 385 | 7.8 | 3.0 | 356 | 6.9 | 2.5 | 314 |  |
| Low-touch right | 8.2 | 3.4 | 409 | 8.8 | 3.4 | 408 | 7.7 | 2.8 | 373 | 6.4 | 3.3 | 334 |  |
| Lunge right | 7.1 | 2.7 | 71 | 7.7 | 3.4 | 68 | 6.5 | 2.8 | 68 | 6.9 | 3.0 | 58 |  |
| Punch right | 9.1 | 3.0 | 707 | 8.6 | 3.2 | 704 | 7.9 | 3.5 | 717 | 6.9 | 2.6 | 620 |  |

*Note. SD = standard deviation, N = number of observations*

Table 3: descriptive means, standard deviation, and number of observations of males and females per side and neutral exercise

| Maximal knee internal rotation angle [°] | Females | | | | | | Males | | | | | | | |
| --- | --- | --- | --- | --- | --- | --- | --- | --- | --- | --- | --- | --- | --- | --- |
|  | Left | | | Right |  |  | Left | | | | Right | | | |
| Exercise | Mean | SD | N | Mean | SD | N | Mean | SD | N | Mean | | SD | N |  |
| Squat | 6.95 | 7.15 | 572 | 4.33 | 6.01 | 594 | 3.89 | 4.94 | 497 | 3.01 | | 6.41 | 579 |  |
| Jump | 10.32 | 6.01 | 741 | 7.66 | 4.49 | 7.20 | 6.95 | 4.97 | 628 | 6.60 | | 5.45 | 734 |  |
| Burpee | 13.10 | 4.96 | 123 | 8.79 | 4.60 | 162 | 7.92 | 3.84 | 97 | 6.76 | | 4.75 | 105 |  |

*Note. SD = standard deviation, N = number of observations*

Table 4: descriptive means, standard deviation, and number of observations of males and females per side and side-specific exercise

| Maximal knee internal rotation angle [°] | Females | | | | | | Males | | | | | |
| --- | --- | --- | --- | --- | --- | --- | --- | --- | --- | --- | --- | --- |
|  | Ipsilateral | | | Kontralateral | | | Ipsilateral | | | Kontralateral | | |
| Exercise | Mean | SD | N | Mean | SD | N | Mean | SD | N | Mean | SD | N |
| High-touch right | 13.93 | 5.14 | 411 | 13.86 | 6.84 | 414 | 8.56 | 5.11 | 379 | 8.20 | 5.02 | 337 |
| Mid-touch right | 13.19 | 5.11 | 384 | 13.45 | 6.56 | 385 | 6.93 | 4.46 | 356 | 8.20 | 4.76 | 314 |
| Low-touch right | 11.12 | 4.65 | 409 | 12.11 | 7.59 | 408 | 5.92 | 5.35 | 373 | 7.73 | 5.76 | 334 |
| Lunge right | 9.23 | 4.06 | 71 | 12.70 | 7.17 | 68 | 5.80 | 4.60 | 68 | 10.85 | 5.69 | 58 |
| Punch right | 16.88 | 6.1 | 707 | 13.01 | 7.58 | 704 | 8.72 | 5.15 | 717 | 8.57 | 6.38 | 620 |

*Note. SD = standard deviation, N = number of observations*

Table 5: descriptive means, standard deviation, and number of observations of males and females per side and neutral exercise

| Minimal hip flexion angle [°] | Females | | | | | | Males | | | | | |
| --- | --- | --- | --- | --- | --- | --- | --- | --- | --- | --- | --- | --- |
|  | Left | | | Right | | | Left | | | Right | | |
| Exercise | Mean | SD | N | Mean | SD | N | Mean | SD | N | Mean | SD | N |
| Squat | 13.20 | 10.96 | 572 | 13.50 | 11.92 | 594 | 15.06 | 9.42 | 497 | 15.61 | 9.71 | 579 |
| Jump | 5.20 | 8.88 | 741 | 5.04 | 10.02 | 747 | 11.63 | 7.66 | 628 | 12.06 | 7.25 | 733 |
| Burpee | -0.67 | 8.68 | 122 | 1.84 | 10.73 | 161 | 6.43 | 12.02 | 97 | 0.40 | 10.16 | 105 |

*Note. SD = standard deviation, N = number of observations*

Table 6: descriptive means, standard deviation, and number of observations of males and females per side and side-specific exercise

| Minimal hip flexion angle [°] | Females | | | | | | Males | | | | | | |
| --- | --- | --- | --- | --- | --- | --- | --- | --- | --- | --- | --- | --- | --- |
|  | Ipsilateral | | | Kontralateral | | | Ipsilateral | | | | Kontralateral | | |
| Exercise | Mean | SD | N | Mean | SD | N | Mean | SD | N | Mean | | SD | N |
| High-touch right | 9.18 | 10.86 | 411 | 5.29 | 9.88 | 414 | 14.47 | 8.17 | 379 | 9.18 | | 8.78 | 337 |
| Mid-touch right | 10.33 | 11.32 | 384 | 6.91 | 10.08 | 385 | 16.0 | 9.37 | 356 | 10.39 | | 8.31 | 314 |
| Low-touch right | 14.45 | 12.34 | 409 | 9.98 | 10.84 | 408 | 18.01 | 9.86 | 373 | 11.04 | | 8.81 | 334 |
| Lunge right | 11.65 | 12.52 | 71 | -0.51 | 11.00 | 68 | 13.81 | 15.18 | 68 | 1.36 | | 15.91 | 58 |
| Punch right | 12.94 | 10.42 | 707 | 1.52 | 9.24 | 704 | 17.87 | 10.08 | 717 | 5.55 | | 11.22 | 620 |

*Note. SD = standard deviation, N = number of observations*

Table 7: total number of performed exercises per participant.

| Subject ID | Sex (m/f) | Squat [n] | Jump [n] | Burpee [n] | High-touch right [n] | Mid-touch right [n] | Low-touch right [n] | Lunge right [n] | Punch right [n] |
| --- | --- | --- | --- | --- | --- | --- | --- | --- | --- |
| Sub01 | m | 63 | 75 | 14 | 39 | 38 | 41 | 7 | 73 |
| Sub02 | f | 66 | 78 | 16 | 47 | 35 | 46 | 5 | 69 |
| Sub03 | f | 75 | 80 | 22 | 47 | 45 | 46 | 10 | 81 |
| Sub04 | f | 65 | 71 | 18 | 30 | 28 | 32 | 6 | 51 |
| Sub05 | m | 75 | 83 | 21 | 46 | 42 | 45 | 9 | 87 |
| Sub06 | m | 70 | 84 | 15 | 43 | 41 | 44 | 8 | 80 |
| Sub07 | m | 67 | 81 | 18 | 43 | 39 | 46 | 8 | 81 |
| Sub08 | m | 68 | 78 | 19 | 45 | 41 | 43 | 8 | 80 |
| Sub10 | f | 78 | 88 | 18 | 49 | 48 | 51 | 9 | 86 |
| Sub11 | f | 78 | 86 | 22 | 50 | 47 | 53 | 9 | 89 |
| Sub12 | f | 78 | 85 | 20 | 50 | 46 | 47 | 9 | 78 |
| Sub14 | f | 78 | 83 | 20 | 46 | 47 | 48 | 8 | 88 |
| Sub16 | m | 76 | 80 | 19 | 46 | 43 | 41 | 6 | 82 |
| Sub18 | m | 66 | 78 | 17 | 42 | 40 | 46 | 8 | 76 |
| Sub19 | m | 76 | 84 | 18 | 49 | 42 | 42 | 6 | 83 |
| Sub20 | f | 78 | 91 | 14 | 48 | 45 | 46 | 9 | 86 |
| Sub21 | f | 77 | 85 | 22 | 47 | 46 | 47 | 9 | 87 |
| Sub22 | m | 70 | 92 | 19 | 44 | 43 | 45 | 10 | 83 |

Note. m = male, f = female, n = number of observations
